# Overhydration and low serum prealbumin predict peritoneal dialysis-related peritonitis in continuous ambulatory peritoneal dialysis patients

**DOI:** 10.1186/s12882-020-02178-w

**Published:** 2020-11-25

**Authors:** Quyen Dao Bui Quy, Tuan Pham Ngoc Huy, Loc Nguyen Duc, My Pham Van, Dung Nguyen Huu, Toan Nguyen Duy, Tien Tran Viet, Quyet Do, Thang Le Viet

**Affiliations:** 1 Cho Ray Hospital, Ho Chi Minh, Viet Nam; 2 Trung Vuong Hospital, Ho Chi Minh, Viet Nam; 3 An Sinh Hospital, Ho Chi Minh, Viet Nam; 4 University of Medicine Pham Ngoc Thach, Ho Chi Minh, Viet Nam; 5 Bach Mai Hospital, Ha Noi, Viet Nam; 6 Military Hospital 103, Ha Noi, Viet Nam; 7 Vietnam Military Medical University, Ha Noi, Viet Nam

**Keywords:** Continuous ambulatory peritoneal dialysis, Overhydration, Serum prealbumin, Peritoneal dialysis, Peritonitis

## Abstract

**Background:**

In this study, we focused on the role of overhydration (OH) and low serum prealbumin concentration in predicting peritonitis in continuous ambulatory peritoneal dialysis (CAPD) patients over a 3-year period.

**Methods:**

We measured serum prealbumin concentration and OH by body composition monitor in 278 CAPD patients (159 males and 119 females) with a mean age of 46 years and a median peritoneal dialysis (PD) duration of 21 months. Cases of PD-related peritonitis were collected over 3 years.

**Results:**

After the 3-year follow-up, 44 patients were diagnosed with PD-related peritonitis (15.8%). Low education, serum glucose, prealbumin, and OH were independent risk factors for predicting peritonitis over 36 months in CAPD patients. Based on the ROC curve model and Kaplan-Meier analysis, we realized that low prealbumin and high OH were independent predictors of 3-year peritonitis in CAPD patients (Prealbumin: AUC = 0.838, cut-off value = 32.5 mg/dL, Se = 90.9%, Sp = 32.9%; OH: AUC = 0.851, cut-off value = 1.33 L, Se = 79.5%, Sp = 85.5%; and log-rank test *p* <  0.001, respectively).

**Conclusion:**

Overhydration and low serum prealbumin were the independent predictors of PD-related peritonitis in CAPD patients.

## Background

Peritoneal dialysis (PD) is a renal replacement therapy based on infusing a sterile solution into the peritoneal cavity through a catheter, and it provides for the removal of solutes and water using the peritoneal membrane as the exchange surface [1–3]. Infusion and drainage of the solution into the peritoneal cavity can be performed in two ways: manually (continuous ambulatory PD) or machine-assisted PD (automated PD) [1, 2].

Peritonitis is a common serious complication of peritoneal dialysis that results in considerable morbidity, mortality, and health care costs [4–6]. Depending on the underlying causative agent, PD-related peritonitis is complicated by relapse in 3–20% (14% overall), catheter removal in 10–88% (22% overall), and permanent HD transfer in 9–74% (18% overall) of cases [7, 8]. After a single episode of peritonitis, the risks of death due to infection and cardiovascular disease are markedly increased in the first month and continue to remain significantly elevated for up to 6 months afterwards [9]. There are many risk factors for peritonitis in PD patients, including low education, malnutrition, insufficient dialysis, catheter-related peritonitis, etc. [10–12].

PD is the second most common method of renal replacement therapy after maintenance hemodialysis (HD) in Vietnam. Similar to other countries around the world, peritonitis often occurs in patients with peritoneal dialysis. This causes many patients to switch to maintenance hemodialysis. Many studies have shown that overhydration (OH) often complicates the clinical course and is associated with an increased risk of peritonitis in PD patients [13, 14]. Chronic fluid overload is related to an increased risk of cardiovascular morbidity and mortality in peritoneal dialysis patients [15]. Additionally, prealbumin (transthyretin) is a hepatic secretory protein used to assess malnutrition in PD patients, and it is related to inflammation and atherosclerosis. It also has prognostic value for peritonitis and mortality in PD patients [16, 17]. Up to now, there is no research in Vietnam on the roles of excess fluid and nutritional factors in the prognosis of peritonitis in continuous ambulatory PD patients. As a result of the above reasons, we conducted this study to determine the prevalence of peritonitis and the predictive values of OH and low serum prealbumin for PD-related peritonitis in Vietnamese peritoneal dialysis patients.

## Methods

### Study design and setting

There were 426 patients on continuous ambulatory peritoneal dialysis (PD duration > 2 months) in the Department of Nephrology, Cho Ray Hospital, Ho Chi Minh City, Vietnam, from February 2014 to February 2017 (36 months). Of these, patients who were younger than 18 years, dropped out from PD within 90 days, or were on long-term hemodialysis or had chronic renal transplant failure before initiating PD, as well as those with acute illness, significant infection, malignancy, hepatitis virus infection, or peritonitis, before collecting data for the study were excluded. The remaining 278 PD patients were provided informed consent before participation in our study. This study was approved by the ethics review committee of the hospital.

The enrolled patients were treated with stable continuous ambulatory peritoneal dialysis (CAPD) using conventional PD solutions (Dianeal 1.5, 2.5% or 4.25% dextrose; Baxter Healthcare), and Y-sets and twin-bag systems were utilized in all of the PD patients. All patients underwent physical examinations and full lab investigations, including serum prealbumin, OH, and measurement of 24-h urine volume to determine residual kidney function, Kt/V, creatinine clearance (CCr), and the peritoneal equilibration test (PET). For the PET, we calculated a 4 h dialysate-to-plasma ratio of creatinine (D/Pcr). The PET was categorized among 4 levels (H: high; HA: high-average; LA: low-average; and L: low), as developed and described by Twardowski in 1987 [18]. OH was measured using a body composition monitor (BCM, Fresenius). We determined cut-off values for OH and prealbumin based on the ROC curve model. In our study, we divided education into 2 levels: high and low education. The patients with elementary and junior secondary education were defined as low education. Anemia was defined as Hb < 130 g/L in males and < 120 g/L in females according to the WHO (2011).

PD-related peritonitis was diagnosed and noted for 3 continuous follow-up years. PD-related peritonitis was diagnosed based on at least two of the following criteria [19]: (1) abdominal pain or cloudiness of PD effluent; (2) white blood cell count in PD effluent > 100/μL with > 50% polymorphonuclear leukocytes; and (3) a positive culture from PD effluent.

Serum prealbumin concentration was measured using a quantitative electrochemiluminescence *method* (*ECLIA*) at the time of enrollment.

### Statistical analyses

All normally distributed and continuous data are represented as the mean ± standard deviation and have been analyzed using Student’s t-test, one-way analysis of variance, and post hoc Bonferroni comparison. All the nonnormally distributed data are represented as median (25 percentile-75 percentile) and have been analyzed using the Mann-Whitney U test and Kruskal-Wallis test. Categorical data are presented as the frequency with percentage and have been analyzed using the chi-squared test. Cox proportional hazard models was performed to identify the predictor of peritonitis. Receiver operating characteristic (ROC) curves with the area under the curve (AUC) were calculated to predict peritonitis in patients after 3 years’ follow-up. Peritonitis prognosis was assessed using Kaplan-Meier analysis and evaluated by the log-rank test. Statistical analysis was performed using the Statistical Package for Social Science (SPSS) version 20.0 (Chicago, IL, USA). A *p*-value < 0.05 was considered significant.

## Results

Table 1 shows the basic demographics of all study subjects. In our study, the mean age of the entire cohort was 48.61 ± 13.63 years. Additionally, 57.2% of patients were male, 16.9% of patients had diabetes mellitus (DM), the median duration of PD was 21 months, 22.7% of patients had low education, 71.9% of patients had lost residual kidney function, 95.3% of patients had anemia, and 15.8% of patients had peritonitis during the 3-year follow-up, with the ratio of positive bacterial cultures being 27.3% (12/44 patients).

**Table 1:** Comparison of demographic and laboratory characteristics of peritonitis and nonperitonitis groups

Clinical characteristics and laboratory parameters	All patients (***n*** = 278)	Peritonitis (***n*** = 44)	Nonperitonitis (***n*** = 234)	***p***
Age (years)	48.61 ± 13.63	54.57 ± 12.25	47.49 ± 13.61	*0.001*
Number of males (n, %)	159 (57.2)	25 (56.8)	134 (57.3)	0.956
PD duration (month)	21 (10–40.25)	18.5 (8–37.5)	23 (10–41)	0.516
Low education (n, %)	63 (22.7)	27 (61.4)	36 (15.4)	*< 0.001*
Hypertension (n, %)	228 (82)	36 (81.8)	192 (82.1)	0.971
Diabetes mellitus (n, %)	47 (16.9)	23 (52.3)	24 (10.3)	*< 0.001*
BMI				
- Mean	21.16 ± 2.93	22.15 ± 3.43	20.97 ± 2.79	0.036
- < 18.5	46 (16.5)	5 (11.4)	41 (17.5)	
- 18.5–22.9	168 (60.4)	21 (47.7)	147 (62.8)	*0.009*
- ≥ 23	64 (23)	18 (40.9)	46 (19.7)	
- Min – Max	14.4–33.3	15.4–31.2	14.4–33.3	N/A
OH (L)	1.27 ± 0.18	1.49 ± 0.21	1.23 ± 0.14	< 0.001
-24 h urine volume (ml)	180 (130–500)	195 (146.25–637.5)	175 (128.75–500)	0.12
- Loss of RKF (n, %)	200 (71.9)	28 (63.6)	172 (73.5)	0.181
- Preservation of RKF (n, %)	78 (28.1)	16 (36.5)	62 (26.5)	
PET				
- Mean (D4/P)	0.7 ± 0.08	0.73 ± 0.08	0.7 ± 0.08	*0.024*
- H (n, %)	20 (7.2)	6 (13.6)	14 (6)	
- HA (n, %)	175 (62.9)	28 (63.6)	147 (62.8)	0.236
- LA (n, %)	80 (28.8)	10 (22.7)	70 (29.9)	
- L (n, %)	3 (1.1)	0 (0)	3 (1.3)	
Blood urea (mmol/L)	19.29 ± 6.11	19.16 ± 6.89	19.31 ± 5.97	0.88
Creatinine (μmol/L)	772.16 (654.9–955.8)	738.97 (608.43–946.95)	778.8 (657.55–961.99)	0.372
Kt/V	1.98 ± 0.3	2.00 ± 0.31	1.97 ± 0.3	0.597
Total CCr (L/week/1.73m^2^)	62.65 ± 9.3	63.13 ± 9.57	62.57 ± 9.27	0.713
Hemoglobin				
- Mean (g/L)	100.55 ± 16.9	95.98 ± 15.06	101.41 ± 17.12	0.051
- Anemia (n, %)	265 (95.3)	43 (97.7)	222 (94.9)	0.41
WBC (g/L)	6.86 ± 1.48	6.94 ± 1.37	6.85 ± 1.5	0.711
Neutrophils (%)	61.38 ± 8.44	61.29 ± 10.77	61.39 ± 7.96	0.94
hs-CRP (mg/L)	2 (1–4)	3.95 (2–5.27)	2 (1–3.7)	*< 0.001*
Glucose (mmol/L)	4.22 (3.77–4.83)	4.47 (3.94–5.34)	4.16 (3.76–4.79)	*0.024*
Uric acid (μmol/L)	414.63 ± 83.78	427.04 ± 101.02	412.3 ± 80.16	0.285
Na + (mmol/L)	136.93 ± 3.76	136.17 ± 3.38	137.07 ± 3.81	0.145
K+ (mmol/L)	3.67 ± 0.78	3.52 ± 0.58	3.7 ± 0.81	0.164
Ca++ (mmol/L)	2.05 ± 0.29	2.04 ± 0.23	2.05 ± 0.31	0.83
Protein (g/dL)	6.51 ± 0.69	6.45 ± 0.8	6.53 ± 0.67	0.515
Albumin (g/dL)	3.69 ± 0.48	3.36 ± 0.61	3.75 ± 0.42	*< 0.001*
Prealbumin (mg/dL)	34.35 ± 8.49	26.34 ± 5.54	35.86 ± 8.1	*< 0.001*

*PD* Peritoneal Dialysis, *BMI* Body Mass Index, *OH* Overhydration, *PET* Peritoneal Equilibration Test, *H* High, *HA* High-Average, *LA* Low-Average, *L* Low, *WBC* White Blood Cell, *hs-CRP* High-Sensitivity C- Reactive Protein

Per the results in Table 1, in the peritonitis group, the average age was older, the proportions of low education and DM were higher, the average OH and hs-CRP levels were higher, and the serum average albumin and prealbumin concentrations were significantly lower than those in the nonperitonitis group (all *p* <  0.001).

There were many independent factors significantly associated with peritonitis, including low education, glucose, prealbumin, and OH, based on the results of Cox proportional hazard models with *p* <  0.05 (Table 2).

**Table 2:** Adjusted and unadjusted Hazard Ratio and 95% confidence interval for Cox proportional hazard models predicts peritonitis in continuous ambulatory peritoneal dialysis patients

Variable	Crude hazard ratio [95% CI]	***p***-value	Adjusted hazard ratio^**a**^ [95% CI]	***p***- value
Low education	3.455 [1.655–7.17]	*0.001*	3.357 [1.463–7.706]	*0.004*
OH (L)	81.319 [17.685–374.839]	*< 0.001*	74.112 [10.562–520.033]	*< 0.001*
hs-CRP (mg/L)	1.179 [1.016–1.368]	*0.03*	1.168 [0.998–1.367]	0.054
Glucose (mmol/L)	1.214 [1.072–1.375]	*0.002*	1.224 [1.07–1.401]	*0.003*
Albumin (g/dL)	0.612 [0.363–1.032]	0.066	0.634 [0.348–1.156]	0.137
Prealbumin (mg/dL)	0.848 [0.802–0.896]	*< 0.001*	0.847 [0.801–0.895]	*< 0.001*

*PD* Peritoneal Dialysis, *hs-CRP* High-Sensitivity C-Reactive Protein, *OH* Overhydration

^a^ Hazard ratio was adjusted by age, diabetes, BMI, and D4/P

**Fig. 1:**
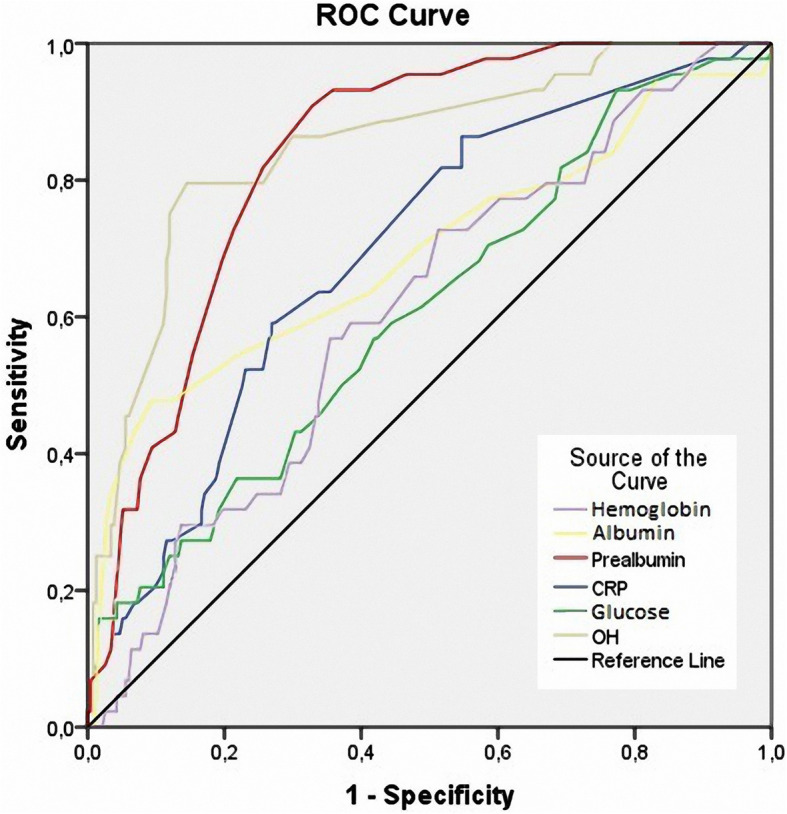
Receiver operating characteristic (ROC) curves of hemoglobin, glucose, hs-CRP, OH, albumin, and prealbumin for the prediction of peritonitis

Based on the results of the ROC curve analysis in Fig. 1, there were many factors predictive for peritonitis, with the prealbumin level and OH having the strongest values (Prealbumin: AUC = 0.838, cut-off value = 32.5 mg/dL, Se = 90.9%, Sp = 32.9%; OH: AUC = 0.851, cut-off value = 1.33 L, Se = 79.5%, Sp = 85.5%).

**Fig. 2:**
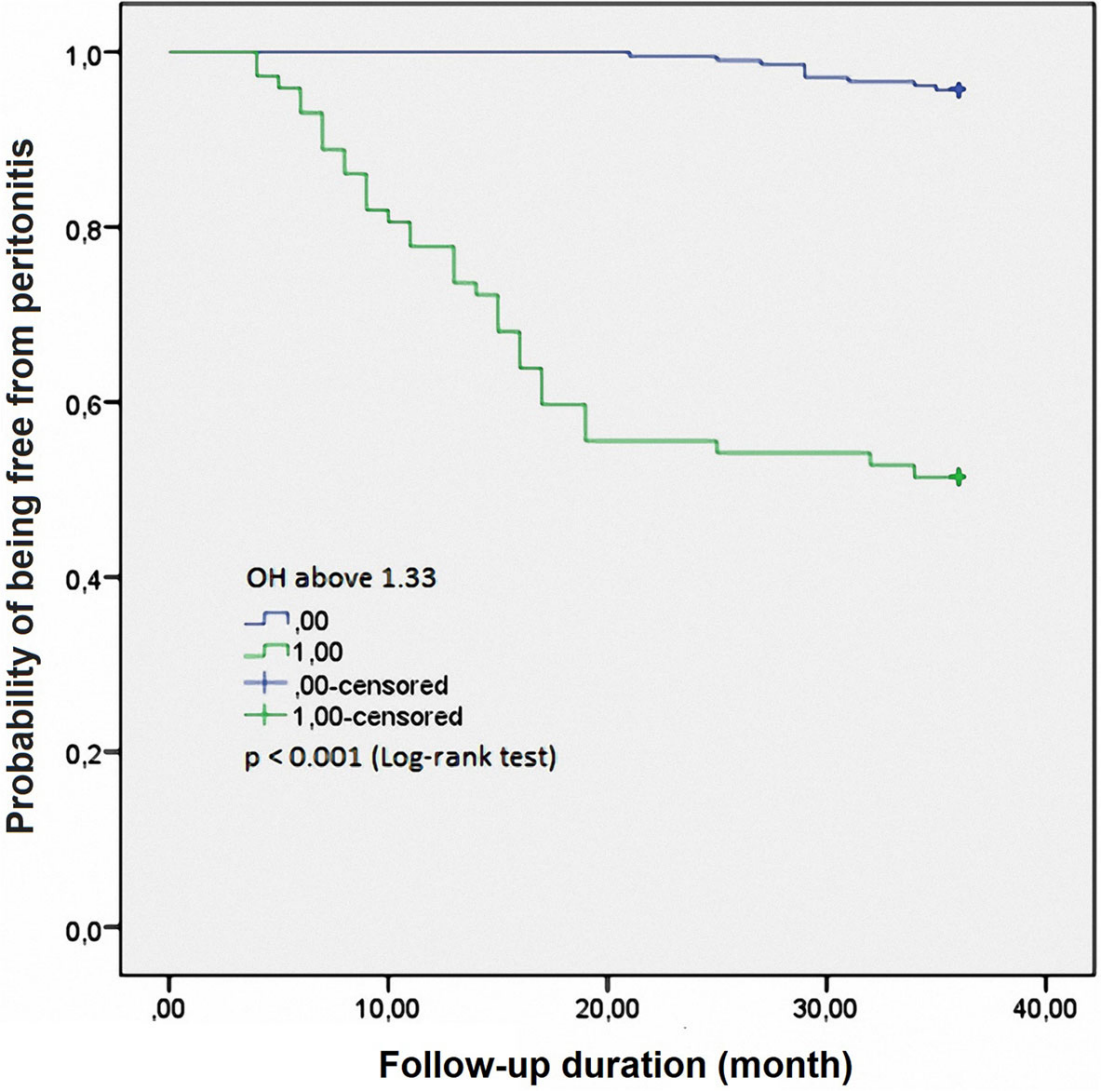
Kaplan-Meier analysis of peritonitis in 278 continuous ambulatory peritoneal dialysis patients, classified according to OH level

The Kaplan-Meier analysis in Fig. 2 shows that patients with high OH (OH ≥ 1.33 L: blue line) exhibit a significantly higher peritonitis rate than those with low OH (OH < 1.33 L: violet line) (log-rank test, *p* <  0.001).

**Fig. 3:**
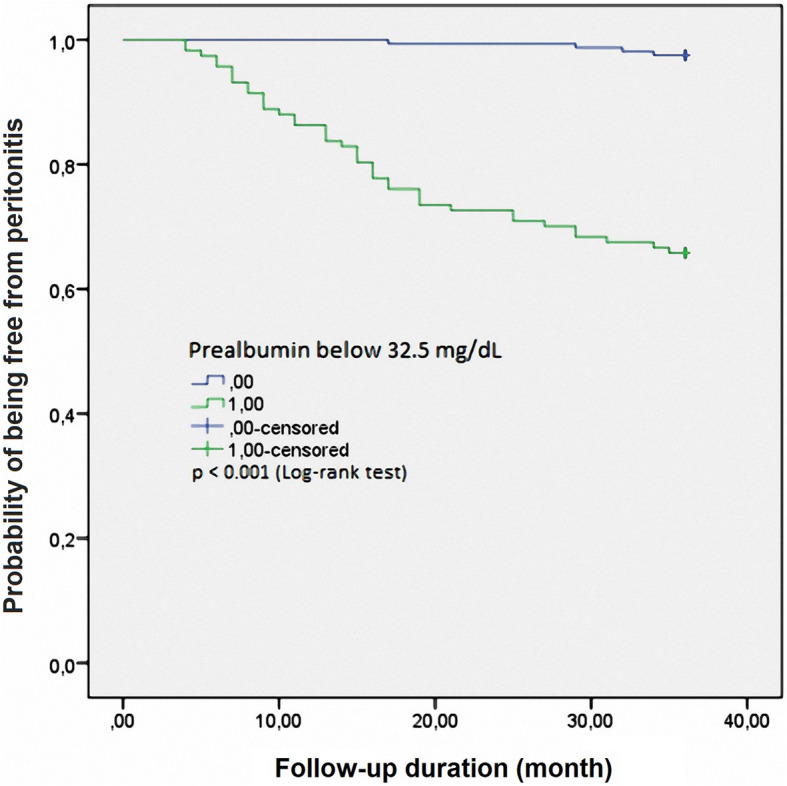
Kaplan-Meier analysis of peritonitis in 278 continuous ambulatory peritoneal dialysis patients, classified according to serum prealbumin concentration

Oppositely, according to the results of Kaplan-Meier analysis in Fig. 3, patients with a low prealbumin level (serum prealbumin ≤32.5 mg/dL: blue line) exhibit a significantly higher peritonitis rate than those with a high serum prealbumin level (serum prealbumin > 32.5 mg/dL: violet line) (log-rank test, *p* <  0.001).

## Discussion

### Prevalence peritonitis

To determine PD-related peritonitis in the end-stage kidney disease patients undergoing CAPD, we excluded patients who had previous peritonitis. After follow-up for 3 years, the proportion of peritonitis in our study was 15.8% (Table 1). There have been some reports about the prevalence of PD-related peritonitis. Ye H. et al. [5] conducted a study with 1321 PD patients with a follow-up of 5 years, and the proportion of peritonitis was 28.16% (372/1321 patients). Furthermore, in the first year of PD initiation, 169 (13%) patients had experienced episodes of peritonitis, and the proportion of patients with peritonitis fluctuated from 8 to 13% in the subsequent years. Gadola L. et al. [20] surveyed the rate of peritonitis in 222 PD patients with a follow-up of 6 years, and the results showed 95 patients suffered 1 or more episodes of peritonitis (42.79%). In a study by Ponce, the proportion of peritonitis in children was 25.45% with a follow-up of 7 years (125 first episodes of peritonitis in 491 PD patients who were children) [21]. The proportion of peritonitis in our study was lower than that in other studies, because our follow-up time was shorter. Overall, 27.3% of patients had a positive bacterial culture in our study, which is similar to other study results [5, 21].

### Relation between peritonitis and some patient characteristics

In Vietnam, peritoneal dialysis is concentrated only in 2 large cities, Hanoi and Ho Chi Minh City. When being recommended chronic kidney replacement therapy, most patients choose maintenance hemodialysis. Only approximately 20% of patients choose peritoneal dialysis, because they do not have time to go to hemodialysis centers. When comparing the peritonitis and nonperitonitis groups, we found some patient characteristics related to peritonitis. In the peritonitis group, the average age was older and the proportions of low education and DM were significantly higher than those in the nonperitonitis group (*p* <  0.001) (Table 1). It remains controversial whether older PD patients have a substantially increased risk of peritonitis than their younger counterparts. More recently, retrospective studies have found that older age (more than 65 years) was the only identifiable risk factor associated with peritonitis [22, 23]. It seems highly probable that touch contamination and bowel dysfunction are important underlying causes of episodes of peritonitis in older PD patients [24]. Diabetes mellitus and low education have been risk factors for PD-related peritonitis in previous studies [24–26]. As diabetes mellitus is regarded a risk factor for infections in general, it seems reasonable to also consider it a risk factor for peritonitis in PD patients [26]. In this study, we found the relationship between peritonitis and overhydration (Tables 1, 2). The results of our study were similar to those of others [13, 14]. The association between OH and peritonitis maybe by enteric microorganisms [14]. This seems to be reasonable, as there is a trend toward an association between baseline levels of C-reactive protein and PD-related peritonitis (Tables 1, 2).

The relationship between peritonitis and malnutrition was also expressed in our study (Tables 1, 2). The average serum albumin and prealbumin levels in the peritonitis group were significantly lower than those in the nonperitonitis group (*p* <  0.001). Peritoneal dialysis itself might lead to protein-energy wasting due to the continuous glucose absorption from peritoneal dialysis solutions and abdominal fullness induced by the dialysate. The result is a decrease in serum albumin and prealbumin concentrations in patients with peritoneal dialysis [27]. Dong J et al. also confirmed that protein leakage predicted the risk for peritonitis in patients on peritoneal dialysis, and this association remained even after adjustment for systemic inflammation estimated by serum albumin, hs-CRP, and IL-6 [28].

### Factors predicting peritonitis

In this study, we found that there were many independent factors related to peritonitis in CAPD patients, of which prealbumin and OH are closely related (*p* <  0.001) (Table 2). We also found that OH and serum prealbumin were the independent predictors of peritonitis compared to other factors, such as glucose, serum albumin, and hs-CRP (AUC of prealbumin was 0.838 and that of OH was 0.851, *p* <  0.001) (Fig. 1). The predictive values, by Kaplan-Meier analysis, for both serum prealbumin and OH with regard to peritonitis were also evident with a follow-up of 3 years (Figs. 2, 3). There are some previous reports about predictive factors for PD-related peritonitis in CAPD patients [20, 22, 23]. Gadola L. et al. [20] confirmed that a multidisciplinary peritoneal educational program improved peritonitis rates, independently of other risk factors. Okayama M. et al. found that aging was an important risk factor for peritoneal dialysis-associated peritonitis [22]. In particular, Kerschbaum J et al. [26] reviewed 415 studies on risk factors for peritonitis in PD patients. From those studies, the author found that the risk factors for peritonitis were divided into two groups: nonmodifiable and modifiable risk factors. Nonmodifiable risk factors are ethnicity, old age, female, cardiovascular comorbidities, DM, underlying renal disease (such as lupus), and loss of residual renal function. Modifiable risk factors are malnutrition, overweight, smoking, comedication with immunosuppressants, depression, and low socioeconomic status. In summary, many risk factors for PD-related peritonitis have been identified in studies of acceptable methodological quality. Overhydration is common among PD patients and related to cardiovascular risk and death [29–31]. Prealbumin levels were an independent and sensitive predictor for mortality in incident PD patients, showing a good correlation with nutritional and inflammatory markers [17, 32]. An association between OH and the risk of peritoneal infection by enteric germs was reported in a study by Carvalho Fiel D [14]. It has been suggested that persistent edema of the intestinal wall may favor microbial and bacterial endotoxin transmigration, leading to systemic infections (including peritonitis) in some cases [14]. Serum albumin and prealbumin are the measures to evaluate the nutritional status of chronic patients in general, especially in patients receiving peritoneal dialysis. Decreased albumin and prealbumin concentrations associated with peritonitis in peritoneal dialysis patients have been mentioned by several authors [26–28]. It might be hypothesized that hypoalbuminemia (as a result of malnutrition, the inflammatory response, or uremia itself) may lead to a higher susceptibility to infection [26]. Thus, both OH and serum prealbumin are modifiable risk factors for PD-related peritonitis, which have predictive value for peritonitis in CAPD patients. This result once again confirms the role of OH and serum prealbumin in predicting outcomes of CAPD patients. From this result, good control of OH and serum prealbumin is needed to reduce the rate of peritonitis in CAPD patients.

Although our results showed that overhydration and low serum prealbumin were the independent predictors of PD-related peritonitis in CAPD patients, this study still had some limitations. First, we did not determine evidence of all other modifiable and nonmodifiable factors related to peritonitis among PD patients [33]. Second, we were unable to obtain repeated measures of prealbumin and OH during the 3-year follow-up period. Therefore, we could not determine the “real” association of prealbumin and OH with the outcome, as well as evaluate the variation of prealbumin and OH in peritoneal dialysis patients over the follow-up time.

## Conclusion

In conclusion, overhydration and low serum prealbumin were the independent predictors of PD-related peritonitis in CAPD patients.

## Data Availability

The datasets used and/or analyzed during the current study are available from the corresponding author on reasonable request.

## Abbreviations

OH: Overhydration
CAPD: Continuous ambulatory peritoneal dialysis
PD: Peritoneal dialysis
CCr: Creatinine clearance
ROC: Receiver operating characteristic
DM: Diabetes mellitus
AUC: Area under the curve

## References

[1] Andreoli MCC, Totoli C (2020). Peritoneal Dialysis. Rev Assoc Méd Bras.

[2] Mehrotra R, Devuyst O, Davies SJ, Johnson DW (2016). The current state of peritoneal dialysis. J Am Soc Nephrol.

[3] Krediet R, Abrahams A, De Fijter C, Betjes M, Boer W, Van Jaarsveld B (2017). The truth on current peritoneal dialysis: state of the art. Neth J Med.

[4] Cho Y, Johnson DW (2014). Peritoneal dialysis–related peritonitis: towards improving evidence, practices, and outcomes. Am J Kidney Dis.

[5] Ye H, Zhou Q, Fan L, Guo Q, Mao H, Huang F (2017). The impact of peritoneal dialysis-related peritonitis on mortality in peritoneal dialysis patients. BMC Nephrol.

[6] Liakopoulos V, Nikitidou O, Kalathas T, Roumeliotis S, Salmas M, Eleftheriadis T (2017). Peritoneal dialysis-related infections recommendations: 2016 update. What is new?. Int Urol Nephrol.

[7] Szeto C-C, Wong TY-H, Chow K-M, Leung C-B, Li PK-T (2003). The clinical course of culture-negative peritonitis complicating peritoneal dialysis. Am J Kidney Dis.

[8] Ghali JR, Bannister KM, Brown FG, Rosman JB, Wiggins KJ, Johnson DW (2011). Microbiology and outcomes of peritonitis in Australian peritoneal dialysis patients. Perit Dial Int.

[9] Boudville N, Kemp A, Clayton P, Lim W, Badve SV, Hawley CM (2012). Recent peritonitis associates with mortality among patients treated with peritoneal dialysis. J Am Soc Nephrol.

[10] Segal JH, Messana JM (2013). Prevention of peritonitis in peritoneal dialysis. Seminars in dialysis.

[11] Bolton L (2019). Preventing peritoneal dialysis infections. Wounds.

[12] Hu S, Ming P, Qureshi AR, Lindholm B, Bo Y, Yang H (2018). Peritonitis: episode sequence, microbiological variation, risk factors and clinical outcomes in a North China peritoneal dialysis center. Kidney Blood Press Res.

[13] Guo Q, Lin J, Li J, Yi C, Mao H, Yang X (2015). The effect of fluid overload on clinical outcome in southern chinese patients undergoing continuous ambulatory peritoneal dialysis. Perit Dial Int.

[14] Carvalho DF, Pérez-Fontán M, López AI, Bravo LG-B, García LG, García TF (2019). Persistent overhydration is associated with a significant risk of peritoneal infection by enteric pathogens in patients treated with peritoneal dialysis. Nefrologia.

[15] Cheng LT, Gao YL, Qin C (2008). Volume overhydration is related to endothelial dysfunction in continuous ambulatory peritoneal dialysis patients. Perit Dial Int.

[16] Fan X, Huang R, Wang J, Ye H, Guo Q, Yi C (2014). Risk factors for the first episode of peritonitis in southern Chinese continuous ambulatory peritoneal dialysis patients. PLoS One.

[17] Lee KH, Cho J-H, Kwon O, Kim S-U, Kim RH, Cho YW (2016). Low prealbumin levels are independently associated with higher mortality in patients on peritoneal dialysis. Kidney Res Clin Pract.

[18] La Milia V (2010). Peritoneal transport testing. J Nephrol.

[19] Li PK-T, Szeto CC, Piraino B, Bernardini J, Figueiredo AE, Gupta A (2010). Peritoneal dialysis-related infections recommendations: 2010 update. Perit Dial Int.

[20] Gadola L, Poggi C, Dominguez P, Poggio MV, Lungo E, Cardozo C (2019). Risk factors and prevention of peritoneal dialysis-related peritonitis. Perit Dial Int.

[21] Ponce D, De Moraes TP, Pecoits-Filho R, Figueiredo AE, Barretti P (2018). Peritonitis in children on chronic peritoneal dialysis: the experience of a large National Pediatric Cohort. Blood Purif.

[22] Okayama M, Inoue T, Nodaira Y, et al. Aging is an important risk factor for peritoneal dialysis-associated peritonitis. Adv Perit Dial. 2012;28:50–4. 23311213

[23] Hsieh Y-P, Chang C-C, Wen Y-K, Chiu P-F, Yang Y (2014). Predictors of peritonitis and the impact of peritonitis on clinical outcomes of continuous ambulatory peritoneal dialysis patients in Taiwan—10 years’ experience in a single center. Perit Dial Int.

[24] Tsai C-C, Lee J-J, Liu T-P, Ko W-C, Wu C-J, Pan C-F (2013). Effects of age and diabetes mellitus on clinical outcomes in patients with peritoneal dialysis-related peritonitis. Surg Infect.

[25] Barretti P, Moraes TM, Camargo CH, Caramori JC, Mondelli AL, Montelli AC (2012). Peritoneal dialysis-related peritonitis due to Staphylococcus aureus: a single-center experience over 15 years. PLoS One.

[26] Kerschbaum J, König P, Rudnicki M. Risk factors associated with peritoneal-dialysis-related peritonitis. Int J Nephrol. 2012;2012:483250. 10.1155/2012/483250 PMC3539329 23320172

[27] Dalrymple LS, Johansen KL, Chertow GM, Grimes B, Anand S, McCulloch CE (2013). Longitudinal measures of serum albumin and prealbumin concentrations in incident dialysis patients: the comprehensive dialysis study. J Ren Nutr.

[28] Dong J, Chen Y, Luo S, Xu R, Xu Y (2013). Peritoneal protein leakage, systemic inflammation, and peritonitis risk in patients on peritoneal dialysis. Perit Dial Int.

[29] Aguiar PV, Santos O, Teixeira L, Silva F, Azevedo P, Vidinha J (2015). Overhydration prevalence in peritoneal dialysis–a 2 year longitudinal analysis. Nefrología (English Edition).

[30] Shu Y, Liu J, Zeng X, Hong HG, Li Y, Zhong H (2018). The effect of overhydration on mortality and technique failure among peritoneal dialysis patients: a systematic review and meta-analysis. Blood Purif.

[31] Oei E, Paudel K, Visser A, Finney H, Fan SL (2016). Is overhydration in peritoneal dialysis patients associated with cardiac mortality that might be reversible?. World J Nephrol.

[32] Mittman N, Avram MM, Oo KK, Chattopadhyay J (2001). Serum prealbumin predicts survival in hemodialysis and peritoneal dialysis: 10 years of prospective observation. Am J Kidney Dis.

[33] Kerschbaum J, König P, Rudnicki M (2012). Risk factors associated with peritoneal-dialysis-related peritonitis. Int J Nephrol.

